# Blunted cardiovascular effects of beta-blockers in patients with cirrhosis: Relation to severity?

**DOI:** 10.1371/journal.pone.0270603

**Published:** 2022-06-28

**Authors:** Puria Nabilou, Karen Vagner Danielsen, Nina Kimer, Jens Dahlgaard Hove, Flemming Bendtsen, Søren Møller

**Affiliations:** 1 Gastro Unit, Medical Division, Copenhagen University Hospital Hvidovre, Hvidovre, Denmark; 2 Department of Cardiology, Copenhagen University Hospital, Hvidovre, Denmark; 3 Department of Clinical Medicine, Copenhagen University Hospital Hvidovre, Hvidovre, Denmark; 4 Dept. Clinical Physiology and Nuclear Medicine, Center for Functional and Diagnostic Imaging and Research, Hvidovre Hospital, Faculty of Health Sciences, University of Copenhagen, København, Denmark; Albert Einstein College of Medicine, UNITED STATES

## Abstract

**Aims:**

Patients with cirrhosis and portal hypertension are at high risk of developing complications such as variceal hemorrhage, ascites, and cardiac dysfunction, the latter of which is known as cirrhotic cardiomyopathy. Since non-selective beta-blockers (NSBB) may aggravate hemodynamic complications we investigated the effect of real-time propranolol infusion on cardiac function in patients with varying degrees of cirrhosis.

**Methods:**

Thirty-eight patients with Child-Pugh A (n = 17), B (n = 17) and C (n = 4) underwent liver vein catheterization and cardiac magnetic resonance imaging. We assessed the effect of real-time propranolol infusion on the hepatic venous pressure gradient, cardiac index, stroke volume, ejection fraction, heart rate, and contractility.

**Results:**

Nineteen patients were classified as responders to beta-blocker therapy. In pooling Child-Pugh B and C patients, the reduction in cardiac index by beta-blockade was weaker than in Child-Pugh A patients (-8.5% vs. -20.5%, *p* = 0.043). The effect of NSBB on portal pressure was inversely correlated to changes in the left atrium where the left atrial volume changed by 4 mL±18 in responders compared to 15 mL±11 in non-responders (*p* = 0.03). Finally, the baseline ejection fraction correlated inversely with the reduction in portal pressure (*r* = -0.39, *p* = 0.02).

**Conclusion:**

We found the effect of beta-blockade on cardiac index in patients with advanced cirrhosis to be less potent than in patients with early cirrhosis, indicating that underlying cirrhotic cardiomyopathy increases, and the cardiac compensatory reserve becomes more compromised, with disease progression. The differential effects of beta-blockade in the left atrium may be used to predict the effect of beta-blockers on portal pressure, but further studies are needed to investigate this possibility.

## Introduction

Patients with advanced cirrhosis are at high risk of developing complications from multiple organ systems, leading to increased morbidity and mortality [1]. Cardiac dysfunction in patients with cirrhosis is described as cirrhotic cardiomyopathy (CCM) and is now widely acknowledged [2]. Ascertaining the presence of CCM is clinically relevant since it appears to be involved in the development of complications in cirrhosis and is associated with the course of the disease [3]. The definition of CCM comprises 1. systolic dysfunction, 2. diastolic dysfunction, and 3. supportive criteria such as heart chamber enlargement, abnormal chronotropic response, and QT-interval prolongation [2].

The exact prevalence of CCM is not known, but estimates range from 50–60% depending on the definition used [4]. It is thus estimated that diastolic dysfunction is present in about 50% of cirrhotic patients depending on the characteristics of the population and the severity of their liver disease [5]. About 50% of patients with cirrhosis present with esophageal varices at the time of diagnosis [6]. Non-selective beta-blockers (NSBB) are well established in primary and secondary prophylaxis of variceal hemorrhage because they reduce portal pressure [7, 8], but only about 50% of patients respond to this therapy [9, 10]. Recently, the potentially deleterious effects of NSBB in decompensated cirrhosis have come under scrutiny [11–14], leading to the formulation of the window hypothesis [15]. This hypothesis states that NSSB have a negative impact on the cardiac compensatory reserve in severe cirrhosis and results in increased mortality [11].

Until now, cardiac changes have primarily been assessed using echocardiography and under strain [2]. Investigating cardiac abnormalities during beta-blockade using cardiac magnetic resonance imaging (CMRI) provides a new opportunity for meticulous assessment of potentially harmful hemodynamic changes. In the present study, we used CMRI, to investigate the effects of acute beta-blockade on cardiac function, as well as changes in portal pressure, among patients with cirrhosis of differing severities.

## Methods

### Study population

In this cross-sectional intervention study, we recruited 38 patients with cirrhosis with a mean age of 59.8±9 years. Seventeen healthy controls with a mean age of 54.2±7 years were included for acquisition of baseline values. The diagnosis of cirrhosis was based on clinical, biochemical, and ultrasonographic indicators. All patients underwent endoscopy that revealed esophageal varices and signs of portal hypertension, with an indication for treatment with NSBB, according to the EASL guidelines [8]: primary prophylaxis in patients with grade 2–3 varices, grade 1 varices with cherry red spot, or Child-Pugh C status with varices, regardless of varix size. Secondary prophylaxis was offered to patients with previous variceal hemorrhaging.

Patients were excluded if they had known heart disease, had undergone a liver transplantation, had had a variceal hemorrhage in the two 2 weeks prior, or a blood transfusion within a week of the study, past insertion of a transjugular intrahepatic portosystemic shunt, hepatic- or portal vein thrombosis, claustrophobia, atherosclerosis, dysregulated insulin dependent diabetes mellitus, acute or chronic renal or cardiovascular disease, any known active cancer, alcoholic hepatitis or a hepatic encephalopathy score higher than 1. The severity of the liver disease was classified according to the Model for End-stage Liver-Disease-Sodium (MELD-Na) and Child-Pugh scores [16].

Of the 38 patients with cirrhosis, 22 were males; 17 patients were categorized as Child-Pugh class A, 17 as class B and four as Child-Pugh class C. Child-Pugh B and C patients were pooled (‘Child-Pugh B/C’) for statistical analysis. Thirty-three patients had alcoholic cirrhosis. The remaining etiologies were non-alcoholic steatohepatitis in two patients (both Child-Pugh A), autoimmune hepatitis in one patient (Child-Pugh B), hepatitis B in one patient (Child-Pugh A) and cryptogenic cirrhosis in one patient (Child-Pugh B). None of the patients with Child-Pugh A had ascites at the time of inclusion, seven patients in the Child-Pugh B/C group had ascites, two of whom were refractory. Two patients each with Child-Pugh A and Child-Pugh B/C, had hypercholesterolemia (>5 mmol/L). Furthermore, one patient in each group had treatment with statins due to hypercholesterolemia. One Child-Pugh B patient had insulin dependent diabetes mellitus, which was well-regulated.

To minimize pharmacological influences on cardiac activity or volume status, diuretics and beta-blocker therapy were paused one and five days, respectively, prior to the investigations. Fasting was required six hours before the hemodynamic investigations in order to reduce interference with changes in preload. All patients provided informed written consent before participating in the study according to the Helsinki Declaration. The trial was approved by the Scientific Ethics Committee of the Capital Region of Denmark (journal no: H-16048475).

### Liver vein catheterization

All patients underwent a liver vein catheterization to diagnose and assess the degree of portal hypertension. The investigations were performed in the morning after an overnight fast and at least one hour of rest in the supine position. Catheterization of the femoral artery and vein were performed under local anesthesia and a small polyethylene catheter was introduced into the femoral artery using the Seldinger technique. A Swan-Ganz catheter was guided through the femoral vein to the hepatic veins during fluoroscopic control. The hepatic venous pressure gradient (HVPG) was measured as the wedged (WHVP) minus the free hepatic venous pressure (FHVP) using a Swan-Ganz balloon catheter [17]. Pressures were measured directly by a capacitance transducer (Simonsen & Weel, Copenhagen, Denmark). Mean arterial pressure (MAP) was determined as described elsewhere [18].

We assessed the response to NSBB according to the Baveno VI criteria, defining it as a reduction in HVPG ≥ 10%, or a HVPG < 12mmHg after an intravenous injection of propranolol at 0.15 mg/kg body weight, up to a maximum dose of 15 mg [19, 20]. After measuring baseline HVPG, the propranolol (Dociton®, MIBE GmbH Arzneimittel, Brehna) was administered as an intravenous five-minute infusion, during which heart rate and blood pressure were monitored. HVPG was measured again 15 minutes after the propranolol infusion. Patients with HVPG less than 10 mmHg were excluded from the study.

### Cardiac magnetic resonance imaging

CMRI recordings were performed with a 1.5 Tesla whole-body scanner (Magnetom Avanto; Siemens Healthineers, Erlangen, Germany) no more than 30 days after patients were recruited to the study and assessed.

To measure diastolic and systolic phases of the cardiac cycle, the long axis (LAX) of the heart was localized to obtain a single-slice, multi-phase, breath-hold ECG-gated sequence. Twenty-five phases per slice were acquired. Both 4-chamber and 2-chamber axial LAX slices were used for biplanar calculation of myocardial morphology as well as global strain [21].

### Cardiac parameters

CVI42 software release 5.9.1 (Circle Cardiovascular Imaging Inc., Calgary, Canada) was used to visualize the endocardial contours of the heart in 4- and 2-chamber views simultaneously in cine-mode. The endocardial contours in all chambers were drawn at the end-diastolic and end-systolic phase in all subjects. The phase with the lowest ventricular volumes and the highest atrial volumes were defined as the end systolic volumes. The end-diastolic volume of the left ventricle was defined as being the phase with the highest ventricular volumes and lowest atrial volumes. As such, the end-diastolic volume was always in phase one, whereas the mean end systolic volume was phase 11 (SD ±1). Left atrial volume was defined in the end-systole. In order to minimize potential bias, all subjects were anonymized prior to delineation.

The left ventricular stroke volume, ejection fraction and cardiac output were automatically calculated based on end-diastolic volume, end systolic volume and heart rate of the patient. Cardiac index was calculated as cardiac output (stroke volume × heart rate in beats per minute (BPM)) divided by the total body surface area, calculated using Du Bois’ formula [22].

A feature-tracking algorithm was applied to the LAX endocardial contour of the left ventricle in both 2-, 3- and 4-chamber views, to assess global longitudinal strain, as well as peak strain values [23]. Endocardial and epicardial contour delineation were performed on the sequence before, and the sequence after, propranolol infusion to calculate differences between global strain values.

After obtaining the baseline sequence, the MR scan was paused and patients were given the propranolol infusion through a peripheral venous catheter located in an antecubital vein, with a dosage of propranolol and timing similar to that of the liver vein catheterization. Systolic blood pressures were monitored with a non-magnetic sphygmomanometer 0, 5, 10 and 15 minutes after infusion to ensure stable blood pressure and heart rate before continuing with the second sequencing. Thus, the patient had to be static and in the supine position for 20 minutes between the two sequences.

### Statistics

Statistical analyses were performed with R-3.6.1 for Windows. All predictor variables were tested for normality with the Shapiro-Wilk test for each group, the total cohort and pooled Child-Pugh B and Child-Pugh C (Child-Pugh B/C). The mean and standard deviation (SD) were reported for parametric variables, while medians with interquartile ranges (IQR) were reported for non-parametric variables. Categorical variables were reported as proportions or percentages. The effect of propranolol on the predictor variables was tested with paired *t*-test or Wilcoxon test, as appropriate, and reported with mean differences (MD) and SD. Differences between multiple groups were compared using ANOVA or the Kruskal-Wallis test, as appropriate. Due to the small sample size of the Child-Pugh C group, Child-Pugh B and C patients were pooled. The comparisons were made with unpaired *t*-test or Mann-Whitney U test, as appropriate. Pearson’s correlation analysis was performed for univariate correlations. *P* values less than 0.05 were considered significant.

## Results

Demographic, clinical, and biochemical characteristics of the cirrhotic patients are shown in Table 1. All patients had portal hypertension with a mean HVPG of 16.8±3.7 mmHg; it was higher in Child-Pugh B/C patients (17.9±4 mmHg) than in Child-Pugh A patients (15.4±2 mmHg) (*p*<0.05). The average MELD-Na and Child-Pugh point-scores were 12.8±4.2 and 7±2, respectively.

**Table 1:** Demographic, clinical, and biochemical characteristics of different Child Pugh groups.

Characteristics	Child-Pugh A (n = 17)	Child-Pugh B/C (n = 21)	*p*
Age	61 ± 8	59 ± 10	0.5
Gender (men/women)	6/11	16/5	0.03
MELD-Na score	11 ± 3	14 ± 5	0.02
Responders	8	11	1
BMI (kg/m2)	27 ± 5	25 ± 5	0.4
Smoking (never/former/current)	2/12/3	4/9/7	-
Hypertension (yes/no)	3/14	4/17	1
Diabetes mellitus (yes/no)	5/12	4/17	0.7
INR	1.2 ± 0.1	1.4 ± 0.2	0.001
Serum-Bilirubin	18 ± 9	29 ± 27	0.1
Serum-Creatinine	75 ± 21	64 ± 18	0.1
Serum-Sodium	137 ± 3	136 ± 4	0.3
Alanine Transaminase (U/L)	38 ± 20	36 ± 12	0.7
Albumin (μmol/L)	39 ± 3	29 ± 6	<0.001
Alkaline phosphatase (U/L)	147 ± 75	156 ± 96	0.8

Data are presented as mean ± SD. Abbreviations: Model for End-Stage Liver Disease-Sodium score (MELD-Na); body mass index (BMI); international normalized ratio (INR). Responders were identified by a reduction in hepatic venous pressure gradient of ≥ 10% or to < 12mmHg after acute beta-blockade.

The mean age of the healthy controls was 54.1±7 years. There was no significant difference in body mass index (BMI) or gender (*p* = 0.4) between the healthy controls and patients.

### Hemodynamic and cardiac parameters at baseline

Cardiac variables, assessed by CMRI, are shown in Table 2 in controls and in Child-Pugh classes A and B/C. The ejection fraction was significantly higher in the cirrhotic patients, than in the controls (0.72±0.08 vs. 0.63 ±0.05) (*p*<0.001). The cardiac index was also higher in the cirrhotic patients than in the controls (3.9 vs. 2.9 L·min^-1^·m^-2^) (*p* = 0.005) showing a strong correlation with MELD-Na (*r* = 0.5, *p* = 0.001). Using Bazett’s formula [24], the QTc-interval was found to be significantly longer in patients than in controls at baseline, and longer in Child-Pugh B/C patients (455 ms [449;468]) than in Child-Pugh A patients (431 ms [420; 440]) (*p* = 0.03). The QTc interval was also found to correlate directly with the MELD-Na score (*r* = 0.60, *p*<0.001). Within the Child-Pugh classes, there were no differences found with respect to baseline MAP or right atrial pressure.

**Table 2:** Cardiac function at baseline and after NSBB in controls vs. Child A vs. BC patients.

Groups	Controls (n = 17)	Child Pugh A (n = 17)		Child Pugh B/C (n = 21)		*p*-values	
	Baseline	Baseline	After NSBB	Baseline	After NSBB	Baseline	After NSBB
End diastolic volume (mL), Left ventricle	146 ± 27	122 ± 26	125 ± 27	138 ± 42	146 ± 38*	0.1	0.07
Stroke Volume (mL)	92 ± 18	89 ± 21	89 ± 21	100 ± 36	103 ± 35	0.4	0.2
Ejection Fraction (%)	63 ± 5	73 ± 9	71 ± 8	72 ± 8	70 ± 9	<0.001	0.6
Cardiac Index (L/min-1*m2)	3 ± 1	3.7 ± 1	2.9 ± 1*	4 ± 1	3.6 ± 1*	0.005	0.022
Heart Rate (BPM)	63 ± 11	80 ± 12	63 ± 8*	80 ± 11	70 ± 13*	<0.001	0.08
Left Atrium (mL)	69 ± 25	60 ± 20	65 ± 29	62 ± 29	74 ± 31*	0.6	0.4
MAP (mmHg)	-	93 ± 13	92 ± 13	91 ± 13	91 ± 13	0.7	0.8
HVPG (mmHg)	-	15 ± 3	13 ± 3*	18 ± 4	16 ± 5*	0.048	0.09
Global Longitudinal Strain (%)	-15.4 ± 2	-15.8 ± 3	-15.7 ± 2	-15.7 ± 3	-15.9 ± 2	0.9	0.8
Time to peak (ms), Longitudinal	323 ± 35	305 ± 41	352 ±64*	294 ± 43	338 ± 39*	0.09	0.06
Peak systolic strain rate (1/s), Longitudinal	-0.79 ± 0.1	-0.85 ±0.1	-0.74 ± 0.1*	-0.86 ± 0.1	-0.79 ± 0.1*	0.3	0.2

*p*-values for parametric data were calculated with ANOVA test, and with the Kruskal-Wallis test for non-parametric data. Data are presented as mean ± SD. Paired comparisons are before/after propranolol infusion in Child-Pugh A and Child-Pugh B/C* *p*<0.05.

Abbreviations: mean arterial pressure (MAP); hepatic venous pressure gradient (HVPG); non-selective beta-blockers (NSBB).

Cardiac morphology at baseline, did not appear to differ significantly between patients and controls, nor between the individual Child-Pugh classes, with respect to left ventricular end-diastolic volume, stroke volume, or size of the left atrium (Table 2). However, there was a direct correlation between the size of the left ventricle (*r* = 0.34, *p* = 0.04) and stroke volume (*r* = 0.34, *p* = 0.03) with an increasing MELD-Na score.

### Impact of beta-blockade on cardiac and hemodynamic parameters

Across the entire patient cohort, NSBB had significant effects on left ventricular end-diastolic volume, cardiac index, heart rate, left atrium size, MAP, longitudinal time to peak, peak systolic strain rate, and HVPG (S1 Table).

**Fig 1:**
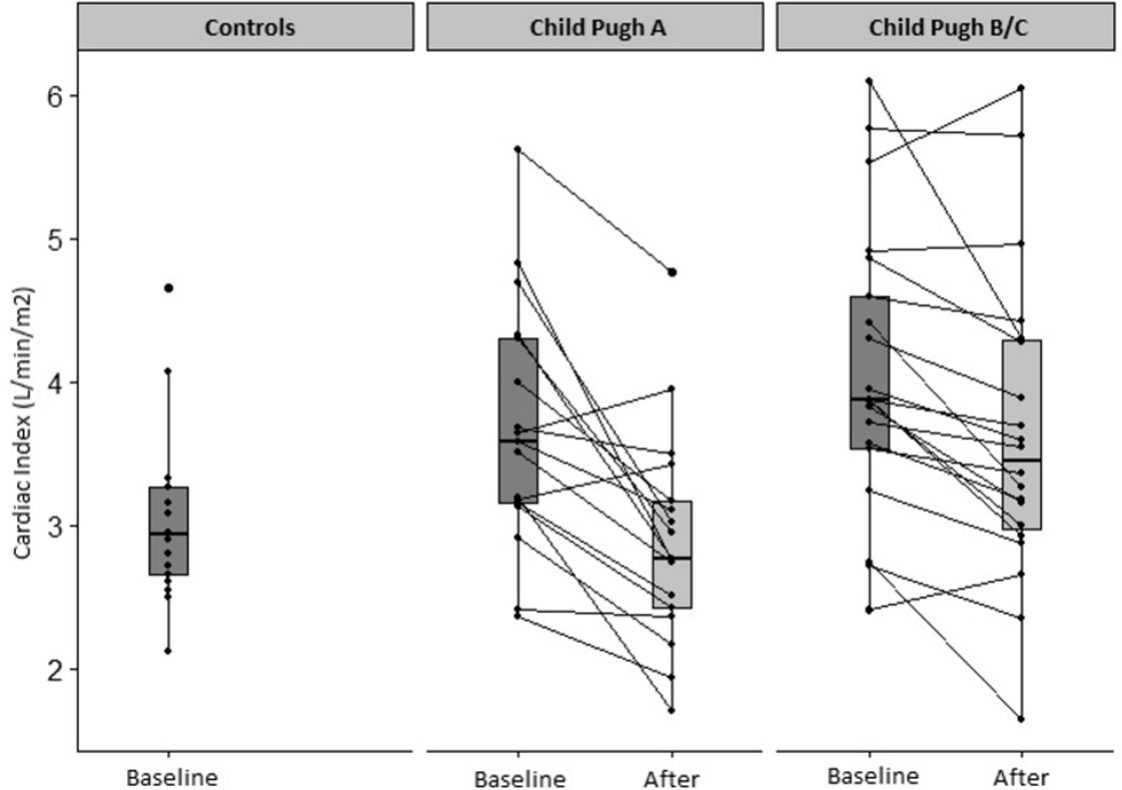
Cardiac index before and after acute beta-blockage. The hyperdynamic syndrome in patients with cirrhosis compared to healthy controls and the effect of beta-blockers on cardiac index among patients with Child-Pugh A (-0.8±0.7 L/min./m^2^ (-20.1%), *p*<0.001) and Child-Pugh B/C (-0.4 L/min./m^2^ (-8.5%), *p* = 0.0013).

**Fig 2:**
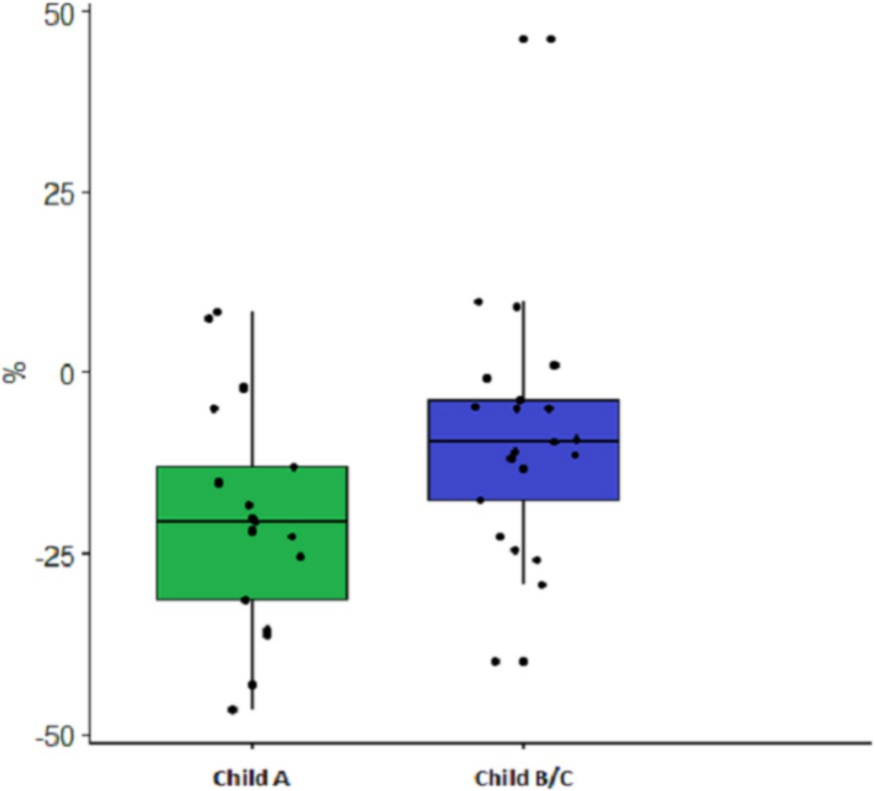
The blunted effect of beta-blockers on cardiac index. The weaker effect of beta-blockers on the cardiac index in Child-Pugh A vs. B/C patients (*p* = 0.043). See also Table 3.

After NSBB, the cardiac index decreased in Child-Pugh A patients by -0.8±0.7 L/min./m^2^ (-20.1%, *p*<0.05). In Child-Pugh B/C patients, the cardiac index decreased by -0.4 L/min./m^2^ (-8.5%, *p*<0.05) (Fig 1). The reduction in cardiac output and index were significantly more pronounced in the Child-Pugh A patients than in the Child-Pugh B/C patients (*p*<0.05), (Fig 2 and Tables 3 and S2).

**Table 3:** The varying impact of NSBB on cardiac parameters in Child-Pugh A vs. B/C patients.

Changes in %	A (n = 17)	BC (n = 21)	*p*
End diastolic volume, Left Ventricle	1.1 [-2; 4]	2.7 [-1; 16]	0.3
Stroke Volume	-3.6 [-8; 5]	1.6 [-5; 11]	0.4
Ejection Fraction	-6.0 [-8; 4]	-1.6 [-8; 3]	0.7
Cardiac Index	-20.1 ± 16	-8.5 ± 18	0.043
Heart Rate	-20.9 ± 12	-12.3 ± 14	0.058
Left Atrium	7.8 ± 24	25.6 ± 31	0.06
MAP	-1.7 ± 4	0.9 ± 6	0.2
HVPG	-14.5 ± 14	-13.1 ± 12	0.7

Data are presented as mean ± SD or medians and interquartile ranges, as appropriate.

Abbreviations: mean arterial pressure (MAP), hepatic venous pressure gradient (HVGP).

In Child-Pugh B/C patients, propranolol had a significant effect on all cardiac parameters other than stroke volume, ejection fraction and strain. Accordingly, there were significant increases in end-diastolic left ventricular volume (MD 8.5 mL±13 (+6.2%), *p* = 0.009) and the left atrium (MD 12 mL ±15 (+19.1%), *p* = 0.002) as well as a significant decrease in heart rate (MD -10 BPM±13 (-12.9%), *p* = 0.001 and cardiac index (MD -0.4±0.6 (-9.2%), *p* = 0.001).

The absolute changes in cardiac index correlated with the absolute change in the left ventricle (*r* = 0.47, *p*<0.01), stroke volume (*r* = 0.62, *p*<0.001), ejection fraction (*r* = 0.46, *p*<0.01), and the change in heart rate (*r* = 0.57, *p*<0.001); there was an inverse correlation with heart rate at baseline (*r* = -0.35, *p*<0.05).

### Varying impact of NSBB between Child-Pugh groups

Comparing Child-Pugh A to Child-Pugh B/C patients, there was a significant difference in the impact of NSBB on cardiac index, with a more powerful reduction in in Child-Pugh A patients (-20.1%±16) than in Child-Pugh B/C patients (-8.5%±18; *p* = 0.043) (Fig 2 and Table 3). Consequently, Child-Pugh B/C patients had a significantly higher cardiac index after beta-blockade than Child-Pugh A patients (3.64±1.1 vs. 2.90±0.8 L/min./m^2^, *p* = 0.022 (Fig 1 and Table 2). It appears that NSBB reduce the heart rate of the Child-Pugh B/C patients less than in Child-Pugh A patients (-10±13 vs. -18±12 BPM, *p* = 0.08) (S2 Table). No difference was found in the effect of NSBB on HVPG in the two groups, although Child-Pugh B/C patients had a higher HVPG than Child-Pugh A at baseline (18±4 mmHg vs. 15±3 mmHg, *p* = 0.048).

### The impact of NSBB in responders and non-responders

Nineteen patients were classified as responders to NSBB, eight patients of whom were Child-Pugh class A, with an average MELD-Na score of 12.5±3; the average MELD-Na of non-responders was 13.4±5 (*p* = 0.5). Two of the non-responders had refractory ascites, as did one responder. Three non-responders and four responders had hypertension (*p* = 1). MAP, HVPG and cardiac volumes were similar at baseline in the two groups. After the beta-blocker test, HVPG was predictably lower in the responders (14 mmHg [10;15]) than in the non-responders (16 mmHg [14;19]) (*p* = 0.002), equivalent to a relative reduction in HVPG of 22.2% [17;28] in the responders vs. 5.9% [0;7] in the non-responders (*p*<0.001). There were no differences found in the cardiac parameters, apart from a higher peak diastolic strain rate in the responders (0.89±0 per. sec) than in non-responders (0.77±0 per. sec) (*p* = 0.04).

**Fig 3:**
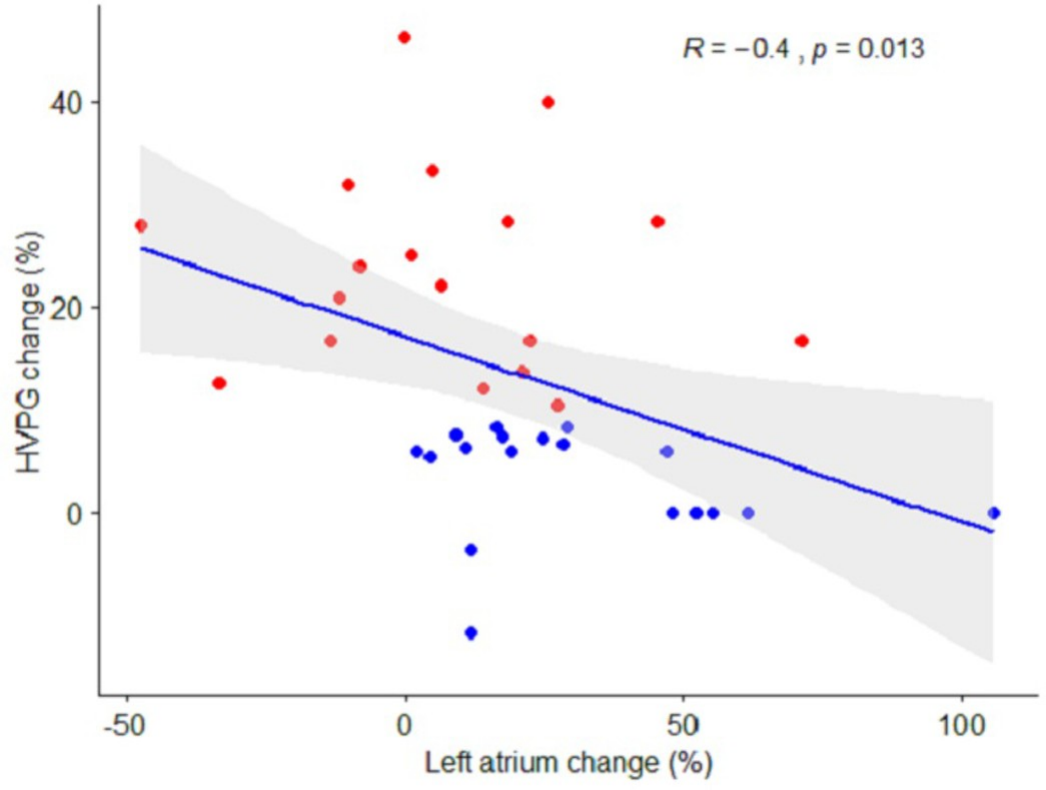
Correlation between changes in portal pressure and size of the left atrium. The impact of beta-blockers on size of the left atrium in relation to the beta-blocker response on portal pressure. The grey area indicates a 95% confidence interval. Red plots: patients classified as responders. Blue plots: patients classified as non-responders.

**Fig 4:**
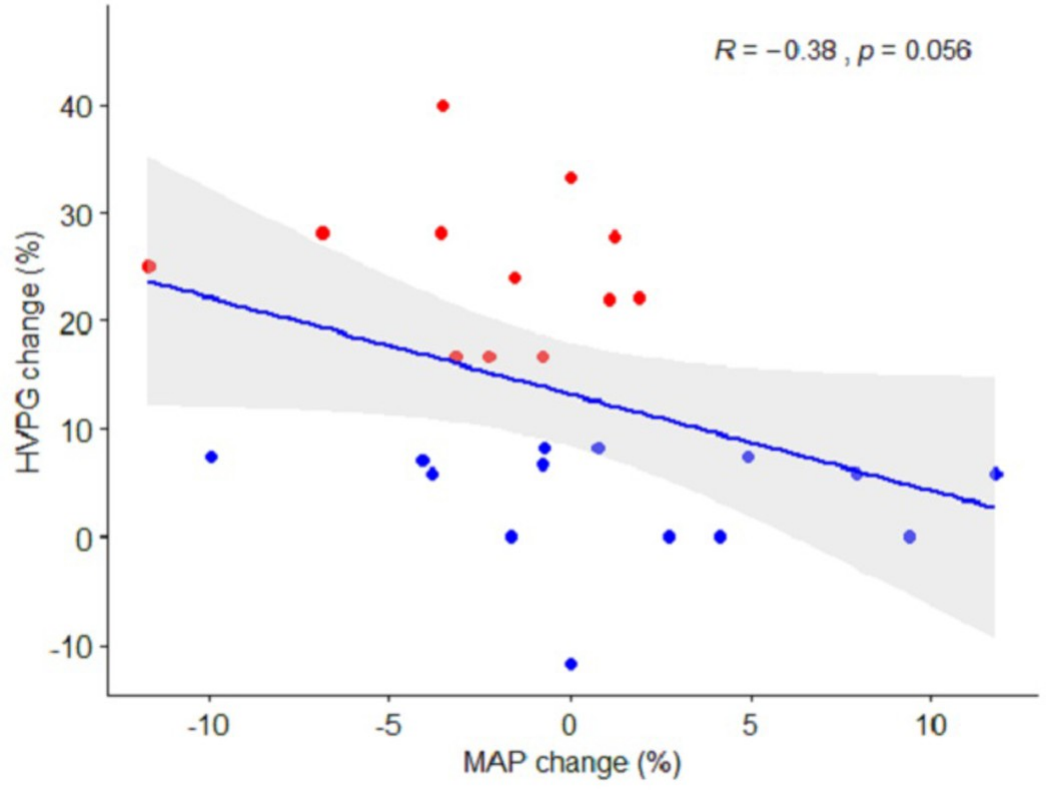
Correlation between changes in portal pressure and mean arterial pressure. The impact of beta-blockers on mean arterial pressure in relation to the beta-blocker response on portal pressure. The grey area indicates a 95% confidence interval. Red plots: patients classified as responders. Blue plots: patients classified as non-responders.

**Fig 5:**
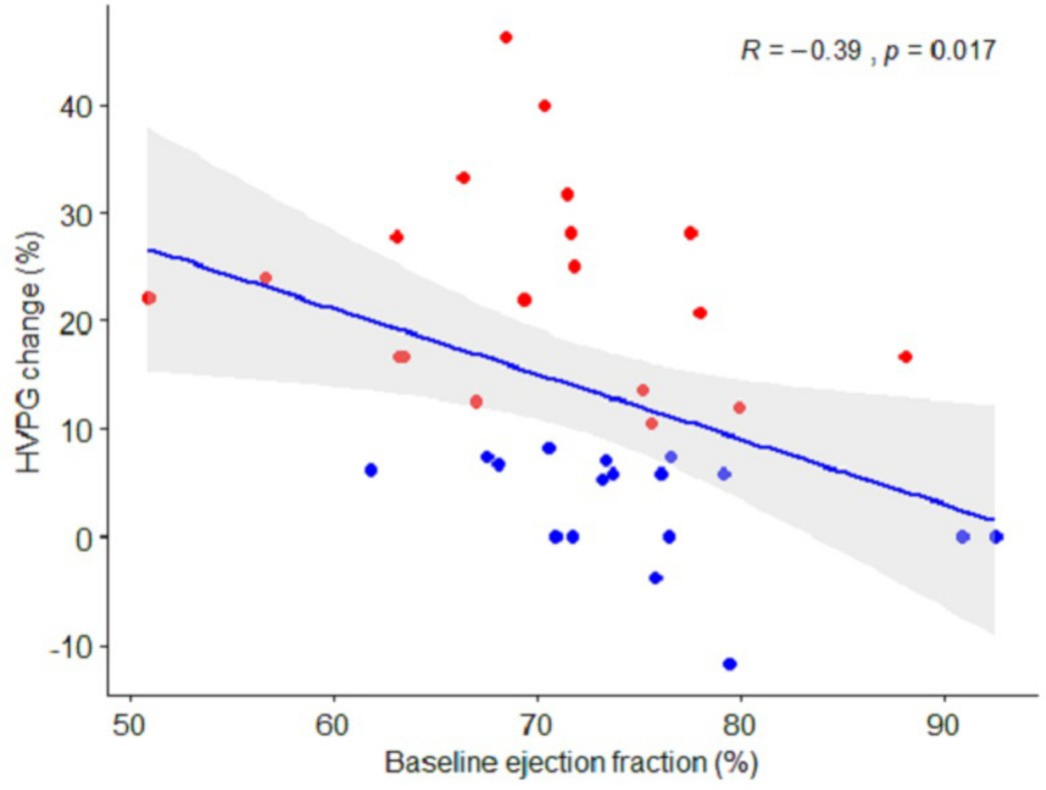
Correlation between changes in portal pressure and baseline ejection fraction. The impact of beta-blockers on baseline ejection fraction in relation to the beta-blocker response on portal pressure. The grey area indicates a 95% confidence interval. Red plots: patients classified as responders. Blue plots: patients classified as non-responders.

The left atrial volume changed by 4 mL±18 in the responders, compared to 15 mL±11 in the non-responders (*p* = 0.03), equivalent to a relative change of 6% [-9; 22] vs. 22% [12;48] (*p* = 0.011) (Table 4). The decrease in HVPG after NSBB was inversely correlated with the increase in left atrial volume (*r* = -0.4, *p* = 0.013, Fig 3). We observed a trend of different effect on MAP with an increase of 1.5% ±6 in the non-responders after NSBB, and a decrease of 2.4% ±4 in the responders (*p* = 0.060). The nominal changes in MAP and HVPG are illustrated in Fig 4. Finally, baseline ejection fraction correlated inversely with the decrease in portal pressure (*r* = -0.39, *p* = 0.02, Fig 5).

**Table 4:** Differing impact of NSBB on cardiac parameters in responders vs. non-responders.

Changes in %	Responders (n = 19)	Non-responders (n = 18)	*p*
End-diastolic volume, Left Ventricle	0.04 [-3; 3]	5.8 [-1; 14]	0.2
Stroke Volume	-2 [-5; 15]	1.8 [-8; 8]	0.8
Ejection Fraction	-0.2 [-7; 4]	-5.2 [-9; 1]	0.2
Cardiac Index	-13.1 [-23; -5]	-16.4 [-25; -6]	0.6
Heart Rate	-15.7 ± 10	-17.9 ± 17	0.6
Left Atrium	6.2 [-9; 22]	21.8 [12; 48]	0.011
MAP	-2.4 ± 4	1.5 ± 6	0.06
HVPG	-22.2 [17; 28]	-5.9 [0, 7]	<0.001

Data are presented as mean ±SD or medians and interquartile ranges as appropriate. Abbreviations: mean arterial pressure (MAP), hepatic venous pressure gradient (HVGP).

## Discussion

In this study, we aimed to investigate the acute effects of beta-blockade on cardiac function using CMRI in patients with varying degrees of cirrhosis. There were two main findings of our study: first, the impact of NSBB on cardiac index was blunted in patients with more severe cirrhosis compared to patients with mild cirrhosis, suggesting that cardiac reserve becomes increasingly compromised as the disease progresses.

Second, in addition to the weaker effect of NSBB on portal pressure, we found that the response to NSBB was inversely correlated with the left atrial volume, which dilated significantly more in non-responders. Both of these findings are most likely the result of an underlying cirrhotic cardiomyopathy (CCM).

A first attempt at defining CCM was made at the World Congress of Gastroenterology in Montreal in 2005, and it continues to be revised as our understanding advances [2]. Most of the studies performed in this area are observational or striving of unmasking the disease by physical or pharmacological stress [25]. The adrenergic signaling system is one of the main regulators of cardiac contractility and vascular tone [26]. It has been one of the main topics of pharmacological studies in patients with cirrhosis since Lebrec discovered that NSBB are able reduce the risk of variceal bleeding by lowering portal pressure [7]. The mechanism of action of NSBB relates to a decrease in cardiac output by the beta-1 adrenoreceptor antagonism and the beta-2 adrenoreceptor antagonistic effect, which causes splanchnic vasoconstriction and thereby reducing in portal pressure. The potentially deleterious effects of NSBB on circulatory function, the development of renal complications, and mortality in severe cirrhosis all continue to be debated [11, 15]. In 2010, Sersté et al. showed that patients with refractory ascites who received treatment with NSBBs had a higher mortality than those who did not [13]. This eventually led to the formulation of the ‘window hypothesis’ that favoured discontinuation of NSBB in patients with end-stage cirrhosis, primarily because of impaired cardiac reserve in these patients [15]. Recently, Téllez et al. identified mechanisms by which NSBB could impair the survival in patients with refractory ascites [12]. These mechanisms relate to systemic vasodilation, a hyperdynamic circulatory state, and increased sympathetic nervous activity, all of which enhance the left ventricular systolic function as a physiological reaction aiming to maintain renal perfusion [27]. From this point of view, the cardiac compensatory reserve is particularly challenged in patients with end-stage cirrhosis, where the acute effects of NSBB on the heart may be blunted as a result of increased sympathetic activity. However, this may not be the case during chronic beta-blocker treatment. Alvarado-tapias, Ardevol et al. (2020) discovered that in the space of 1 to 3 months, the chronic effect of beta-blockers on cardiac output, might adversely influence survival in decompensated cirrhosis, as patients with a marked decrease in cardiac output were more likely to die [28]. These patients did not show an increased cardiac response after acute NSBB administration, which is in line with our findings of a blunted cardiac response to acute beta-blockade in advanced disease.

In a pharmacological study of seventy-one cirrhotic patients with normal left ventricular systolic function at baseline, Kim et al. found that 25% of the patients had blunted left ventricular response during dobutamine infusion [29]. Two studies have tested cardiac function in cirrhotic patients under physical stress. Grose et al. found a reduced capacity for exercise in association with chronotropic incompetence and increased stroke volume mediated by an increased end-diastolic volume [30]. Wong et al. found a significantly smaller increase in the cardiac index of cirrhotic patients than healthy subjects [31]. Both studies found the diastolic and systolic dysfunctions compatible with the condition of CCM. Blunted or abnormal contractile reserve is defined as a failure to increase ejection fraction by 5% in response to stress which can occur in CCM as a potential marker [2]. However, stress testing has limited diagnostic value in clinical practice. In our study, we found that left ventricular ejection fraction decreased after NSBB in patients with mild disease but not in patients with advanced disease (i.e., Child-Pugh B and C patients). This suggests that left ventricular ejection fraction can be used as a marker of impaired cardiac contractile reserve and points to CCM as a contributing factor in patients with advanced disease and therefore NSBB could be used as a diagnostic tool for identifying patients with impaired contractile reserve and at risk for CCM.

The results of previous studies of exercise undertaken by cirrhotic patients indicate that cardiac chronotropic incompetence as part of the cirrhotic cardiac dysfunction and CCM [32, 33]. We found a trend of a blunted reduction in heart rate in patients with advanced disease compared to those with milder disease. Whether this difference could reflect a chronotropic incompetence is possible but is difficult to conclude since we did not perform exercise tests of our patients. Future studies should seek to elucidate whether NSBB can be used to diagnose this type of cardiac dysfunction in cirrhosis. Emerging studies support the use of the non-selective beta-blocker carvedilol, in low doses, as it seems safe to use in both compensated and decompensated cirrhosis [34]. This is particularly interesting from a cardiovascular point of view, as carvedilol, in addition to the aforementioned beta-1 and beta-2 effect, also has an intrinsic alpha-1 inhibitory effect antagonizing the effect of norepinephrine, thus reducing intrahepatic vascular tone [35]. Since we found that NSBB did not affect stroke volume or ejection fraction, the effects of propranolol on cardiac output are primarily achieved by a reduction in heart rate, and can therefore most likely be attributed to differences in the cardiac beta-1 receptors in cirrhosis [36]. A number of experimental studies have shown decreased beta-adrenergic receptor density and receptor desensitization in cardiomyocytes of cirrhotic rats [37, 38]. In a clinical study, Laffi et al. showed that cirrhotic patients had an impaired autonomic cardiovascular response to tilting, despite an appropriate response of increased plasma norepinephrine, which points to a post-receptor defect of autonomic dysfunction [39].

Our findings of an inadequate response to the increased sympathetic nervous activity, in terms of a blunted response to NSBB, favors the hypothesis of an autonomic dysfunction as an imposing part of CCM rather than a general deconditioning; however, a full understanding of the mechanisms of cardiac chronotropic incompetence will depend on future research.

Interestingly, the cardiac index of some patients increased after beta-blockage. Paradoxical effects of NSBB have previously been observed and described in patients with increased sympathetic drive and emotional stress [40, 41]. Since patients with advanced disease often present with elevated sympathetic drive [42], this may explain the paradoxical reaction seen in our study but again, further studies are needed to explore this phenomenon in patients with cirrhosis.

In patients with advanced disease, such as refractory ascites, low cardiac index and a dilated left atrium have proved to be strong prognostic indicators of poor survival [33, 43, 44]. Our results support the understanding of a dilated left atrium as a major cardiocirculatory event since it appeared to predict the impact of NSBB on portal pressure in a group comparison. In a multivariate analysis we have previously shown significant associations between the level of total bile acids and atrial volume [45]. Bile acids exert cardio-suppressive effects on the myocardium and may explain the link between cholestasis and cardiac dysfunction. Similarly, bilirubin, which is also included in the MELD-Na and Child-Pugh scoring systems, possess significant prognostic value. Our observation that serum bilirubin increases with the severity of disease supports the assumption of increased bilirubin as a contributor to the blunted response on cardiac performance in cirrhosis [45, 46].

Arterial blood pressure is regulated by several physiological mechanisms, which in cirrhosis counteract arterial vasodilatation. It is therefore unsurprising that there was no significant difference in arterial blood pressure between NSBB responders and non-responders (*p* = 0.060). Yet interestingly, the change in arterial blood pressure did correlate with the change in portal pressure (*r* = 0.39, *p* = .047). Furthermore, we observed that a high ejection fraction at baseline was associated with a smaller decrease in portal pressure (*r* = -0.39, *p* = 0.017). One possible explanation for this is that NSSB responders exhibit less desensitization to beta-2 receptors than non-responders [47].

In this study we, assessed the acute effects of NSBB on cardiac performance and their relationship to disease severity. Beta-blocker dosage was calculated according to the body weight of patients and primarily for assessing the portal pressure response [20]. Future studies of patients with cirrhosis should seek to modulate the dosage of NSBB while measuring cardiopulmonary hemodynamics, along with assessment of neurohumoral factors and preferably in patients at later stages of cirrhosis, when NSBB can impair cardiovascular homeostasis. To differentiate and elucidate the role of adrenergic signaling in CCM, prospective studies should investigate the impact of differences between selective beta 1- and beta 2-blockade, along with non-selective beta- and alpha-blockade.

### Limitations

Using CMRI to assess the hemodynamic response to NSBB has not previously been undertaken among well-characterized patients with cirrhosis and portal hypertension. Our image analyses were completely blinded and CMRI is considered an accurate and reproducible method. However, due to the restrictive exclusion and inclusion criteria, the results of our study are limited by the relatively small cohort and few patients with advanced disease. Although CMRI is non-invasive, it is costly, and assessments of volume are associated with interobserver variation. While CMRI is considered the gold standard for contractility-measurements [48], measurements of cardiac output are more precisely determined with the thermodilution method [49]. The gap-in time between the CMRI sequences may have had an impact on the chamber delineations and thus the calculations of cardiac output. Our protocol did not allow myocardial fibrosis and extracellular volumes to be assessed.

The etiology of cirrhosis in most of our patients was due to excessive alcohol consumption. The precise influence of alcohol abuse or liver disease related effects on cardiac dysfunction are difficult to distinguish and thus our results should be validated in larger studies that include patients with non-alcoholic etiologies.

Finally, while we accept that using the left atrium as a predictor for the response to beta-blockers is speculative, we do believe that our data are sufficiently encouraging to warrant further studies exploring this phenomenon.

## Conclusion

We found a blunted effect of beta-blockade on cardiac performance in patients with advanced cirrhosis compared to those with mild disease. In addition, the size of the left ventricle and left atrium increased with disease progression indicating impaired compensatory cardiac reserve. The differential effects of beta-blockade on the left atrium may predict the effect of beta-blockers on portal pressure and the prognostic value of this observation should be further investigated in novel studies utilizing combined modalities.

## Supporting information

S1 Table Paired tests on impact of NSBB in patients with cirrhosis (n = 38). Data was tested for normality with Shapiro-Wilks test and students paired t-test or Wilcoxon-signed rank test was used as appropriate for calculation of p-values. Data are presented as mean ±SD. Abbreviations: Mean Arterial pressure (MAP), Hepatic venous pressure gradient (HVGP), Non-selective betablockers (NSBB).
(PDF)

S2 Table Impact differences of NSBB on cardiac parameters between Child A vs. BC patients. Data are presented as mean ±SD or medians and interquartile ranges. Abbreviations: Mean Arterial pressure (MAP), Hepatic venous pressure gradient (HVGP).
(PDF)

S3 Table Impact differences of NSBB on cardiac parameters between responders vs. non-responders. Data are presented as mean ±SD or medians and interquartile ranges as appropriate. Abbreviations: Mean Arterial pressure (MAP), Hepatic venous pressure gradient (HVGP).
(PDF)

## References

[1] Jepsen P., Vilstrup H., and Andersen P. K., “The Clinical Course of Cirrhosis: The Importance of Multistate Models and Competing Risks Analysis,” Hepatology, vol. 62, no. 1, pp. 292–302, 2015, doi: 10.1002/hep.27598 25376655

[2] Izzy M. et al., “Redefining Cirrhotic Cardiomyopathy for the Modern Era,” Hepatology, vol. 71, no. 1, pp. 334–345, 2020, doi: 10.1002/hep.30875 31342529 PMC7288530

[3] Lee S. S., “Cardiac abnormalities in liver cirrhosis,” West. J. Med., vol. 151, no. 5, pp. 530–535, 1989. 2690463 PMC1026787

[4] Razpotnik M. et al., “Prevalence of cirrhotic cardiomyopathy according to the old and new diagnostic criteria,” J. Hepatol., vol. 73, no. March, pp. S740–S741, 2020, doi: 10.1016/s0168-8278(20)31930-9

[5] Møller S., Wiese S., Halgreen H., and Hove J. D., “Diastolic dysfunction in cirrhosis,” Heart Fail. Rev., vol. 21, no. 5, pp. 599–610, 2016, doi: 10.1007/s10741-016-9552-9 27075496

[6] Garcia-Tsao G. et al., “Prevention and management of gastroesophageal varices and variceal hemorrhage in cirrhosis,” Hepatology, vol. 46, no. 3, pp. 922–938, 2007, doi: 10.1002/hep.21907 17879356

[7] Lebrec D., “Proranolol for prevention of recurretn GI Bleeding in patients with Cirrhosis (NEJM—1981),” N. Engl. J. Med., pp. 1371–1374, 1981. 10.1056/NEJM198112033052302 7029276

[8] Angeli P. et al., “EASL Clinical Practice Guidelines for the management of patients with decompensated cirrhosis,” J. Hepatol., vol. 69, no. 2, pp. 406–460, 2018, doi: 10.1016/j.jhep.2018.03.024 29653741

[9] Villanueva C. et al., “β blockers to prevent decompensation of cirrhosis in patients with clinically significant portal hypertension (PREDESCI): a randomised, double-blind, placebo-controlled, multicentre trial,” Lancet, vol. 393, no. 10181, pp. 1597–1608, 2019, doi: 10.1016/S0140-6736(18)31875-0 30910320

[10] Feu F. et al., “Relation between portal pressure response to pharmacotherapy and risk of recurrent variceal haemorrhage in patients with cirrhosis,” Lancet, vol. 346, no. 8982, pp. 1056–1059, 1995, doi: 10.1016/s0140-6736(95)91740-3 7564785

[11] Ge P. S. and Runyon B. A., “Treatment of patients with cirrhosis,” N. Engl. J. Med., vol. 375, no. 8, pp. 767–777, 2016, doi: 10.1056/NEJMra1504367 27557303

[12] Téllez L. et al., “Non-selective beta-blockers impair global circulatory homeostasis and renal function in cirrhotic patients with refractory ascites,” J. Hepatol., pp. 1–11, 2020, doi: 10.1016/j.jhep.2020.05.011 32446716

[13] Sersté T. et al., “Deleterious effects of beta-blockers on survival in patients with cirrhosis and refractory ascites,” Hepatology, vol. 52, no. 3, pp. 1017–1022, 2010, doi: 10.1002/hep.23775 20583214

[14] Sersté T. et al., “Beta-blockers cause paracentesis-induced circulatory dysfunction in patients with cirrhosis and refractory ascites: A cross-over study,” J. Hepatol., vol. 55, no. 4, pp. 794–799, 2011, doi: 10.1016/j.jhep.2011.01.034 21354230

[15] Krag A., Wiest R., Albillos A., and Gluud L. L., “The window hypothesis: Haemodynamic and non-haemodynamic effects of β-blockers improve survival of patients with cirrhosis during a window in the disease,” Gut, vol. 61, no. 7, pp. 967–969, 2012, doi: 10.1136/gutjnl-2011-301348 22234982

[16] Kim W. R. et al., “Hyponatremia and mortality among patients on the liver-transplant waiting list,” N. Engl. J. Med., vol. 359, no. 10, pp. 1018–1026, 2008, doi: 10.1056/NEJMoa0801209 18768945 PMC4374557

[17] Hobolth L., Bendtsen F., and Moller S., “Indications for portal pressure measurement in chronic liver disease,” Scand. J. Gastroenterol., vol. 47, no. 8–9, pp. 887–892, 2012, doi: 10.3109/00365521.2012.706827 22809270

[18] Møller S., Hobolth L., Winkler C., Bendtsen F., and Christensen E., “Determinants of the hyperdynamic circulation and central hypovolaemia in cirrhosis,” Gut, vol. 60, no. 9, pp. 1254–1259, 2011, doi: 10.1136/gut.2010.235473 21504996

[19] de Franchis R. and Faculty Baveno VI, “Expanding consensus in portal hypertension: Report of the Baveno VI Consensus Workshop: Stratifying risk and individualizing care for portal hypertension.,” J. Hepatol., vol. 63, no. 3, pp. 743–52, Sep. 2015, doi: 10.1016/j.jhep.2015.05.022 26047908

[20] Villanueva C. et al., “Acute Hemodynamic Response to β-Blockers and Prediction of Long-term Outcome in Primary Prophylaxis of Variceal Bleeding,” Gastroenterology, vol. 137, no. 1, pp. 119–128, 2009, doi: 10.1053/j.gastro.2009.03.048 19344721

[21] Ito H. et al., “Cardiovascular magnetic resonance feature tracking for characterization of patients with heart failure with preserved ejection fraction: Correlation of global longitudinal strain with invasive diastolic functional indices,” J. Cardiovasc. Magn. Reson., vol. 22, no. 1, pp. 1–11, 2020, doi: 10.1186/s12968-020-00636-w 32498688 PMC7271439

[22] Du Bois D. and Du Bois E. F., “A formula to estimate the approximate surface area if height and weight be known. 1916.,” Nutrition, vol. 5, no. 5, p. 303, 1989. 2520314

[23] Scatteia A., Baritussio A., and Bucciarelli-Ducci C., “Strain imaging using cardiac magnetic resonance,” Heart Fail. Rev., vol. 22, no. 4, pp. 465–476, 2017, doi: 10.1007/s10741-017-9621-8 28620745 PMC5487809

[24] Bazett H. C., “An analysis of the time-relations of electrocardiograms.,” Ann. Noninvasive Electrocardiol., vol. 2, no. 2, pp. 177–194, Apr. 1997, doi: 10.1111/j.1542-474X.1997.tb00325.x

[25] Krag A., Bendtsen F., Dahl E. K., Kjær A., Petersen C. L., and Møller S., “Cardiac Function in Patients with Early Cirrhosis during Maximal Beta-Adrenergic Drive: A Dobutamine Stress Study,” PLoS One, vol. 9, no. 10, pp. 11–13, 2014, doi: 10.1371/journal.pone.0109179 25279659 PMC4184863

[26] Santulli G., “Sympathetic nervous system signaling in heart failure and cardiac aging,” in Pathophysiology and Pharmacotherapy of Cardiovascular Disease, 2015.

[27] Yotti R. et al., “Left ventricular systolic function is associated with sympathetic nervous activity and markers of inflammation in cirrhosis,” Hepatology, vol. 65, no. 6, pp. 2019–2030, 2017, doi: 10.1002/hep.29104 28195341

[28] Alvarado-Tapias E. et al., “Short-term hemodynamic effects of β-blockers influence survival of patients with decompensated cirrhosis,” J. Hepatol., vol. 73, no. 4, pp. 829–841, 2020, doi: 10.1016/j.jhep.2020.03.048 32298768

[29] Kim M. Y. et al., “Dobutamine stress echocardiography for evaluating cirrhotic cardiomyopathy in liver cirrhosis.,” Korean J. Hepatol., vol. 16, no. 4, pp. 376–382, 2010, doi: 10.3350/kjhep.2010.16.4.376 21415581 PMC3304603

[30] Grose R. D. et al., “Exercise-induced left ventricular dysfunction in alcoholic and non-alcoholic cirrhosis,” J. Hepatol., vol. 22, no. 3, pp. 326–332, 1995, doi: 10.1016/0168-8278(95)80286-x 7608484

[31] Wong F., Girgrah N., Graba J., Allidina Y., Liu P., and Blendis L., “The cardiac response to exercise in cirrhosis,” Gut, vol. 49, no. 2, pp. 268–275, 2001, doi: 10.1136/gut.49.2.268 11454805 PMC1728392

[32] Ruiz-del-Arbol L. et al., “Systemic, Renal, and Hepatic Hemodynamic Derangement in Cirrhotic Patients with Spontaneous Bacterial Peritonitis,” Hepatology, vol. 38, no. 5, pp. 1210–1218, 2003, doi: 10.1053/jhep.2003.50447 14578859

[33] Krag A., Bendtsen F., Henriksen J. H., and Moøller S., “Low cardiac output predicts development of hepatorenal syndrome and survival in patients with cirrhosis and ascites,” Gut, vol. 59, no. 1, pp. 105–110, 2010, doi: 10.1136/gut.2009.180570 19837678

[34] Rodrigues S. G., Mendoza Y. P., and Bosch J., “Beta-blockers in cirrhosis: Evidence-based indications and limitations,” JHEP Reports, vol. 2, no. 1, pp. 1–17, 2020, doi: 10.1016/j.jhepr.2019.12.001 32039404 PMC7005550

[35] Ciccarelli M., Sorriento D., Coscioni E., Iaccarino G., and Santulli G., “Adrenergic Receptors,” in Endocrinology of the Heart in Health and Disease: Integrated, Cellular, and Molecular Endocrinology of the Heart, 2017.

[36] Alexanderl G. Gerbes; Dieter Jüngst; Jörg Remien; Tilman Sauerbruch; Paumgartner, “Evidence for down-regulation of beta-2-adrenoceptors in cirrhotic patients with severe ascites. The Lancet.” The Lancet, p. Vol.327(8495), pp.1409–1411, 1986. 10.1016/s0140-6736(86)91556-4 2872517

[37] Ma Z., Miyamoto A., and Lee S. S., “Role of altered β-adrenoceptor signal transduction in the pathogenesis of cirrhotic cardiomyopathy in rats,” Gastroenterology, vol. 110, no. 4, pp. 1191–1198, 1996, doi: 10.1053/gast.1996.v110.pm8613009 8613009

[38] Ceolotto G. et al., “An abnormal gene expression of the β-adrenergic system contributes to the pathogenesis of cardiomyopathy in cirrhotic rats,” Hepatology, vol. 48, no. 6, pp. 1913–1923, 2008, doi: 10.1002/hep.22533 19003918

[39] Laffi G. et al., “Impaired cardiovascular autonomic response to passive tilting in cirrhosis with ascites,” Hepatology, vol. 24, no. 5, pp. 1063–1067, 1996, doi: 10.1053/jhep.1996.v24.pm0008903376 8903376

[40] “Unexpected pressor responses to propranolol in essential hypertension. An interaction between renin, aldosterone and sympathetic activity,” Am. J. Med., vol. 60, no. 6, p. A24, 1976, doi: 10.1016/0002-9343(76)90911-6 190882

[41] Cleophas T. J. M. and Kauw F. H. W., “Pressor Responses from Noncardioselective Beta-Blockers,” Angiology, vol. 39, no. 7, pp. 587–596, 1988, doi: 10.1177/000331978803900706 2900614

[42] Henriksen J. H., Møller S., Ring-Larsen H., and Christensen N. J., “The sympathetic nervous system in liver disease,” J. Hepatol., vol. 29, no. 2, pp. 328–341, 1998, doi: 10.1016/s0168-8278(98)80022-6 9722218

[43] Merli M., Torromeo C., Giusto M., Iacovone G., Riggio O., and Puddu P. E., “Survival at 2 years among liver cirrhotic patients is influenced by left atrial volume and left ventricular mass,” Liver Int., vol. 37, no. 5, pp. 700–706, 2017, doi: 10.1111/liv.13287 27782364

[44] Cesari M., Frigo A. C., Tonon M., and Angeli P., “Cardiovascular predictors of death in patients with cirrhosis,” Hepatology, vol. 68, no. 1, pp. 215–223, 2018, doi: 10.1002/hep.29520 28902431

[45] Voiosu A. M., Wiese S., Voiosu T. A., Hove J., Bendtsen F., and Møller S., “Total bile acid levels are associated with left atrial volume and cardiac output in patients with cirrhosis,” Eur. J. Gastroenterol. Hepatol., vol. 30, no. 4, pp. 392–397, 2018, doi: 10.1097/MEG.0000000000001043 29227330

[46] Voiosu A., Wiese S., Voiosu T., Bendtsen F., and Møller S., “Bile acids and cardiovascular function in cirrhosis,” Liver Int., vol. 37, no. 10, pp. 1420–1430, 2017, doi: 10.1111/liv.13394 28222247

[47] Zacharias A. P. et al., “Carvedilol versus traditional, non‐selective beta‐blockers for adults with cirrhosis and gastroesophageal varices,” Cochrane Libr., vol. 2018, no. 10, pp. CD011510–CD011510, 2018, doi: 10.1002/14651858.CD011510.pub2 30372514 PMC6517039

[48] Lima J. A. C. and Desai M. Y., “Cardiovascular magnetic resonance imaging: Current and emerging applications,” J. Am. Coll. Cardiol., vol. 44, no. 6, pp. 1164–1171, 2004, doi: 10.1016/j.jacc.2004.06.033 15364314

[49] Kelly C. R. and Rabbani L. R. E., “Pulmonary-artery catheterization,” N. Engl. J. Med., vol. 369, no. 25, p. e35(1), 2013, doi: 10.1056/NEJMvcm1212416 24350972

