# Supplementary material for: Blunted cardiovascular effects of beta-blockers in patients with cirrhosis: Relation to severity?
Source: PLoS One. 2022 Jun 28;17(6):e0270603. doi: 10.1371/journal.pone.0270603 (PMC9239488; doi:10.1371/journal.pone.0270603)
Supplement: S1 Table — Data was tested for normality with Shapiro-Wilks test and students paired t-test or Wilcoxon-signed rank test was used as appropriate for calculation of p-values. Data are presented as mean ±SD. Abbreviations: Mean Arterial pressure (MAP), Hepatic venous pressure gradient (HVGP), Non-selective betablockers (NSBB). (PDF) [file pone.0270603.s001.pdf]

**Patients (n=38)**	**Before NSBB**	**After NSBB**	**p**
End diastolic volume (mL), Left Ventricle	131 (36)	137 (35)	0.022
Stroke Volume (mL)	95 ± 30	97 ± 30	0.4
Ejection Fraction (%)	72 ± 8	70 ± 9	0.09
Cardiac Output (L/min.)	7 ± 2	6 ± 2	< 0.001
Cardiac Index (L/min.*m^2)	3.8 ± 1	3.3 ± 1	< 0.001
Heart Rate (BPM)	80 ± 11	67 ± 12	< 0.001
Left Atrium (mL)	61 ± 25	70 ± 30	0.002
MAP (mmHg)	92 ± 13	92 ± 13	0.6
HVPG (mmHg)	17 ± 4	15 ± 4	<0.001
Global Longitudinal Strain (%)	-15.7 ± 3	-16 ± 2	0.7
Time to peak (ms), longitudinal	299 ± 42	344 ± 51	< 0.001
Peak systolic strain rate (1/s), longitudinal	-0.85 ± 0.1	-0.77 ± 0.1	0.002
