# Supplementary material for: Blunted cardiovascular effects of beta-blockers in patients with cirrhosis: Relation to severity?
Source: PLoS One. 2022 Jun 28;17(6):e0270603. doi: 10.1371/journal.pone.0270603 (PMC9239488; doi:10.1371/journal.pone.0270603)
Supplement: S2 Table — Data are presented as mean ±SD or medians and interquartile ranges. Abbreviations: Mean Arterial pressure (MAP), Hepatic venous pressure gradient (HVGP). (PDF) [file pone.0270603.s002.pdf]

Absolute Changes	Child-Pugh A (n=17)	Child-Pugh B/C (n=21)	p
End diastolic volume (mL), Left ventricle	1.2 [-2.6, 5.3]	4.3 [-1.7, 20]	0.3
Stroke Volume (mL)	0.2 ± 13	2.8 ± 13	0.5
Ejection Fraction (%)	-4.4 [-6.1, 2.8]	-1.2 [-6.4, 2.2]	0.8
Cardiac Output (L./min.)	-1.5 ± 1.4	-0.7 ± 1.2	0.047
Cardiac Index (L/min.*m^2)	-0.8 ± 0.7	-0.4 ± 0.6	0.06
Heart Rate (BPM)	-18 ± 12	-10 ± 13	0.08
Left Atrium (mL)	4.6 ± 17	12 ± 15	0.2
MAP (mmHg)	-2.50 ± 6.5	0.64 ± 7.8	0.3
HVPG (mmHg)	-2.19 ± 2.2	-2.24 ± 2.0	0.9
