# Supplementary material for: Blunted cardiovascular effects of beta-blockers in patients with cirrhosis: Relation to severity?
Source: PLoS One. 2022 Jun 28;17(6):e0270603. doi: 10.1371/journal.pone.0270603 (PMC9239488; doi:10.1371/journal.pone.0270603)
Supplement: S3 Table — Data are presented as mean ±SD or medians and interquartile ranges as appropriate. Abbreviations: Mean Arterial pressure (MAP), Hepatic venous pressure gradient (HVGP). (PDF) [file pone.0270603.s003.pdf]

**Absolute Changes**	**Responder (n=19)**	**Non-Responder (n=18)**	**p**
End diastolic volume (mL), Left ventricle	0.06 [-4; 5]	7.0 [-1; 14]	0.2
Stroke Volume (mL)	2.1 ± 13	1.6 ± 14	0.9
Ejection Fraction (%)	-0.2 [-5; 3]	-3.8 [-7; 1]	0.2
Cardiac Output (L./min.)	-0.95 ± 1	-1.26 ± 1	0.5
Cardiac Index (L/min.*m^2)	-0.5 ± 0.7	-0.6 ± 0.7	0.5
Heart Rate (BPM)	-13 ±10	-15 ±15	0.8
Left Atrium (mL)	4 ± 18	15 ± 11	0.03
MAP (mmHg)	-4 ± 6	2 ± 8	0.07
HVPG (mmHg)	-3.5 [3; 5]	-1 [0; 1]	<0.001
